# SNPExpress: integrated visualization of genome-wide genotypes, copy numbers and gene expression levels

**DOI:** 10.1186/1471-2164-9-41

**Published:** 2008-01-25

**Authors:** Mathijs A Sanders, Roel GW Verhaak, Wendy MC Geertsma-Kleinekoort, Saman Abbas, Sebastiaan Horsman, Peter J van der Spek, Bob Löwenberg, Peter JM Valk

**Affiliations:** 1 Department of Hematology, Erasmus University Medical Center, Rotterdam, The Netherlands; 2 Department of Medical Oncology, Dana-Farber Cancer Institute, Harvard Medical School, Boston, USA; The Broad Institute of M.I.T. and Harvard, Cambridge, USA; 3 Department of Bioinformatics, Erasmus University Medical Center, Rotterdam, The Netherlands

## Abstract

**Background:**

Accurate analyses of comprehensive genome-wide SNP genotyping and gene expression data sets is challenging for many researchers. In fact, obtaining an integrated view of both large scale SNP genotyping and gene expression is currently complicated since only a limited number of appropriate software tools are available.

**Results:**

We present SNPExpress, a software tool to accurately analyze Affymetrix and Illumina SNP genotype calls, copy numbers, polymorphic copy number variations (CNVs) and Affymetrix gene expression in a combinatorial and efficient way. In addition, SNPExpress allows concurrent interpretation of these items with Hidden-Markov Model (HMM) inferred Loss-of-Heterozygosity (LOH)- and copy number regions.

**Conclusion:**

The combined analyses with the easily accessible software tool SNPExpress will not only facilitate the recognition of recurrent genetic lesions, but also the identification of critical pathogenic genes.

## Background

High-density genome-wide views of biological samples, using high-throughput DNA mapping and mRNA gene expression microarrays facilitate the identification of recurrent molecular lesions. Both types of microarrays, which are being produced by different manufacturers, e.g., Nimblegen, Agilent, Sequenom, Applied Biosystems, Illumina and Affymetrix, typically contain large numbers of small oligonucleotides that interrogate the genome. Currently available DNA arrays contain over 500.000 probe sets, while the gene expression arrays target over 20.000 genes. Efficient analysis of these large datasets remains a challenge for many researchers.

The Affymetrix and Illumina DNA mapping platforms have been designed to specifically target sequences containing single nucleotide polymorphisms (SNPs). SNPs are currently estimated to be present at a frequency of 1 out of 300 nucleotides [1]. By including different probe sets to detect the possible SNP variants, genome-wide genotyping is feasible. In fact, these types of arrays have been developed for genome-wide association studies; however, these platforms can easily be applied to determine copy numbers of these chromosomal markers, similar to array comparative genomic hybridization (CGH). Because of the high number of SNPs, sample DNA can be examined with an inter-marker distance of 6 to 12 kb, and (micro) deletions and/or amplifications are detectable. By comparing disease samples to normal germ line DNA, a detailed overview of acquired gains and losses of the genome is obtained. In fact, although our knowledge is still developing, it has recently become apparent that that copy number variation (CNV) accounts for a substantial amount of genetic variation in the human genome [2]. The high-resolution scanning technologies enable the analyses of CNV and associated phenotypes [2].

The power of DNA mapping has been shown extensively in cancer research. Chromosomal gains and losses as well as regions of loss-of-heterozygosity (LOH) have been shown in, for instance, leukemia [3,4], lung cancer [5-7] and colon cancer [8]. Recognition of recurrent lesions will ultimately result in the identification of pathogenic genes. For instance, SNP array analysis of a set of cancer cell lines has lead to the identification of the microphthalmia-associated transcription factor *MITF *as a melanoma oncogene [9].

On the Illumina platform genotypes are determined using hybridization of genomic DNA to BeadChips followed by an enzymatic discrimination step. On the Affymetrix platform, genotype calls and copy numbers are determined by a probe set consisting of mismatch and perfect match probes. In analogy with the expression probe set, the genotype and copy number of an individual SNP is dependent on the balance of genotype calls in the associated probe set. Several methods for genotype calling [10-13] and assessment of copy number [14,15] have been developed. Advanced analysis methods of DNA mapping array data have focused on the identification of regions of LOH, or gains and losses [16-19].

A particular SNP genotype or a numerical change in chromosome copy number can have profound effects on gene expression. A possible relation to tumor development was shown in breast cancer, where a 17q23 amplification was related to increased expression of genes at that locus [20] and in acute myeloid leukemia (AML), where amplification of 8p24 was associated with increased expression of genes such as *MYC *[21]. In fact, SNPs as well as CNVs have recently been shown to have consistent effects, often in *cis*, on gene expression [22,23]. The integrated analysis of gene expression and SNP array data is a prerequisite to recognize these effects. To our knowledge, only one software package is able to visualize chromosome copy number and gene expression levels [17]. Here, we present a package, SNPExpress, which allows concurrent interpretation of genotype, HMM inferred LOH regions, copy number, CNVs, HMM inferred copy number and gene expression data. Due to the simple format of the input data, our package is not restricted to specific methods to determine genotype, copy numbers or expression level. Little knowledge of software is necessary to use SNPExpress, making the tool accessible for a wide audience.

## Implementation

SNPExpress, written in JAVA (version 1.5), uses tab-delimited files as input and is currently available for use with Affymetrix DNA mapping arrays (10 K 2.0, 100 K set and 500 K set), Illumina HumanHap550 Genotyping BeadChip and Affymetrix GeneChips (HG-U95Av2, HG-U133A and B, HG-U133 plus 2.0). A file containing a matrix with each column representing the genotypes of one array and rows starting with Illumina or Affymetrix SNP IDs is mandatory. The genotype should be formatted as homozygous 'AA' or 'BB', heterozygous 'AB', or, 'noCall' (Affymetrix)/'NC' (Illumina). Similar matrix files containing copy numbers or gene expression values are optional. Copy numbers should be centered around 2, where 2 represents the normal copy number of the autosomes and 1 for the male X chromosome. The maximum displayed copy number is 4, in case the copy number is above 4 this is indicated by the greyblue background. Copy number-, genotype- and gene expression files required for SNPExpress can be generated through tools such as Affymetrix BRLMM [13], GCOS/CNAT 4.0 [24], or dChipSNP [17] with additional formatting in Microsoft Excel. In case of Illumina data, SNP Express includes the non-synonymous SNPs and the MHC region, however, mitochondrial SNPs and Y-chromosome SNPs are not visualized. All files can be optionally uploaded as tab- or comma-delimited .txt files or binary files. These binary files can be created from .txt files by the menu item 'convert data source'.

SNPExpress maps both the SNP IDs (Illumina and Affymetrix) and the expression probe set IDs (Affymetrix) to the genome through internal alignment tables, using annotation provided by the manufacturer [25,26] and [27]. Annotation was generated using NCBI build 36.1.

Regions showing LOH are calculated through a hidden Markov Model, which has been described previously [18]. The probability values for heterogeneous calls required for the HMM have been generated through sets of genotypes of normal samples. For the 100 K and 500 K array, 90 samples and 270 samples, respectively, of different ethnical background from the HapMap project are available through the NCBI GEO website (and provided by the manufacturer) [28,29]. For the 10 K array normal matched blood samples available through the GEO public repository have been processed [30]. Since reference normal Illumina genotype datasets are currently not publicly available, LOH regions using this platform are not supported in this version of SNPExpress.

SNPExpress includes the option to visualize the results of a novel analytical method that infers the copy number of each SNP based on a HMM model, which is implemented in dChipSNP [17,31]. Also, all CNVs [2], currently cataloged in the Database of Genome Variants [32], can be visualized.

Example expression, copy number, genotype and HMM copy number example files of two AML patients can be downloaded from [33].

## Results

**Figure 1:**
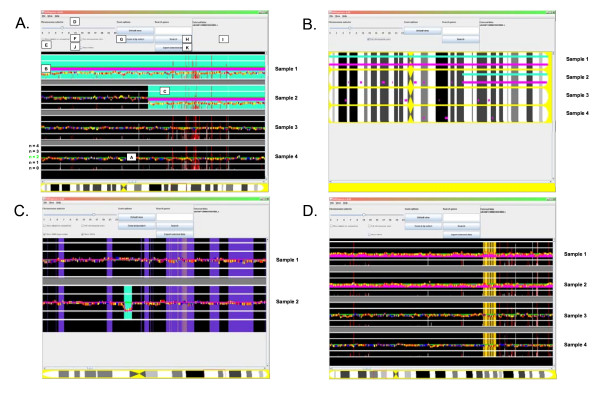
SNPExpress Screenshot. **A**. DNA mapping array data from the Affymetrix 250 K *Nsp*I DNA mapping array was used to sequentially align the genotypes and copy numbers of chromosome 7 of four AML samples. The copy numbers (n = 0, 1, 2, 3, 4) are shown for each individual patient by horizontal lines. Copy number n = 2 is depicted by a green line (A). The SNP genotypes are sequentially aligned along the chromosome (AA: red; BB: yellow; AB: blue, noCall: white). LOH is indicated by a thick magenta horizontal bar (B), gains (default n > 2.5) by a pink (Figure 1C) and losses (default n < 1.5) by a turquoise background (C). Gene expression levels are visualized as vertical white bar at the chromosomal position of the gene-specific probe set. In the event that multiple probe sets span the same region in the chromosome-wide view the vertical gene expression bars are red and proportional to the highest expression value. The two upper samples clearly display a decreased copy number as was previously shown by cytogenetics, i.e., a complete monosomy (sample 1) or a deletion of the q-arm of chromosome 7 (sample 2). The overall expression of the majority of genes in the displayed region is decreased in the samples with chromosome 7 abnormalities. The chromosome selector (D; where 23 is the X chromosome), the mouse-over function showing info of each SNP or probe set (E), full chromosome view (F), zoom function (G) gene search function (H), the links to external databases (I), display CNVs (J) and export selected data (K) options are indicated. **B**. Full chromosome view of samples from 1A. **C**. CNV (purple background) and copy number of each SNP based on a HMM model (HMM copy number, magenta line) of the two AML patients from examples [33]. In the event that multiple CNVs span the same region in the chromosome-wide view the background is violet, whereas single CNV are indicated with a rosy brown background. **D**. UPD of chromosome 11 demonstrated using SNPExpress. Example of large scale UPD on chromosome 11 in the upper two AML patients with a normal karyotype in comparison to two other AML samples. The overall copy number is two and large regions of LOH are indicated by the thick magenta line across the chromosome. After using the search function, SNPs associated with *WT1 *are depicted with an orange background.

Genotypes and copy numbers are displayed as sequential blocks of which color indicates genotype, horizontal coordinate indicates position on the chromosome and vertical coordinate indicates copy number (Figure 1). The colored genotype blocks are drawn sequential in chromosome-wide view and proportional to chromosomal location when zoomed into a region of interest. Gene expression levels are visualized as vertical bar at the chromosomal position of the gene-specific probe set. The height of the bar is proportional to the gene expression value. The default value is 500 and expression higher than 500 is capped at 500, however, these values are user-definable. In the event that multiple probe sets span the same region in the chromosome-wide view the vertical gene expression bars are red and proportional to the highest expression value. Zooming into the location of interest discloses the individual probe sets. Links of SNP IDs to public databases are available by holding the ctrl-key and clicking on a SNP ID.

Distinct background colors are used to accentuate genomic changes. Individual copy numbers are indicated as gain (pink background) or loss (green background) when their value exceeds a user-defined value. The default deviation threshold is 0.5. LOH is highlighted at diploid level by a bold magenta line (Figure 1). All colors can be adapted to the users' preferences.

From the menu, the user is able to choose to visualize either one chromosome of multiple samples or the complete genome of one sample. Detailed information, containing information such as SNP ID, associated gene symbol, probe set ID, cytoband and expression value, is shown on a mouse-over display. Furthermore, a gene of interest is directly visualized through a search function, and its associated SNPs are indicated with an orange background color. The options to display known CNVs (purple background) or the HMM copy number results (thin magenta line) are included (Figure 1C). Finally, relevant data of a particular minimal deleted of amplified region can be exported (i.e. Sample, Probe_set_id, Chromosome, Location (bp), Cytoband, Associated gene, Genotype, Copy number and Inferred LOH of the selected region) and high-resolution images of the visualization can be saved in the Portable Network Graphic (PNG) format.

To illustrate the power of SNPExpress, DNA mapping array profiles of tumor samples of a series of 48 patients with AML were generated using Affymetrix 250 K *Nsp*I DNA mapping arrays. Ficoll separation of the mononuclear cells from AML typically yields >80% pure population of leukemic blast cells. High molecular weight DNA was isolated from these malignant cells and the Affymetrix mapping arrays were used according to the protocol of the manufacturer. Genotypes were calculated using BRLMM and copy numbers were assessed using dChipSNP. Biotin-labeled cRNA of the same AML samples was hybridized on Affymetrix HG-U133 plus 2.0 GeneChips, as described elsewhere [34]. The resulting dataset was imported in SNPExpress for analyses. Large chromosomal regions showing loss or gains of genetic material are known to be apparent in leukemic blasts of AML patients. Well-known examples of chromosomal lesions in AML are monosomies of chromosome 5 and 7, which have been associated with a poor prognosis [35]. Using SNPExpress, monosomies of chromosome 7 were evidently demonstrated in AML samples, previously shown by cytogenetics to have lesions involving chromosome 7 (Figure 1). SNPExpress also correctly predicted the presence of LOH as a result of the absence of one chromosome 7. In fact, 17 out of 21 numerical cytogenetic aberrations, i.e., whole chromosomes and interstitial deletions, in 48 AML samples analyzed, were recognized by using SNPExpress. Four numerical abnormalities abnormalities, present in less than 30% of the AML cells, were missed. Chromosomal gains, losses as well as uniparental disomy (UPD) may also have other important consequences, such as affecting expression of (imprinted) genes. Combinatorial visualization of genotype, copy number and gene expression is a prerequisite to recognize these aberrations. For example, the majority of genes show located on chromosome 7 show an overall decrease in expression in AML samples with a monosomy 7 (Figure 1).

Large regions of homozygosity are present in approximately 20% of primary AML cases as a result of segmental UPD [3,36]. These regions of UPD seemed to be non-random and may be used to unmask pre-existing recessive mutations in leukemia genes, such as *CEBPA*, *WT*1, *FLT3 *and *RUNX*1 [3,37]. SNPExpress adequately identified regions of UPD involving e.g. chromosome 11p (Figure 1D), in two patients with a normal karyotype. UPD involving chromosome 11 is associated with homozygous mutations in *WT1 *[37]. Interestingly, in 13 out of 48 AML patients (27%) large regions of segmental UPD continuing to the telomere were recognized using SNPExpress.

These examples demonstrate the power of SNPExpress. To our knowledge, no tool is currently available that allows concurrent interpretation of genotype, HMM inferred LOH regions, copy number, CNVs, HMM inferred copy number and gene expression data. Moreover, no specialized knowledge is necessary to work with SNPExpress.

## Discussion

Since genome-wide DNA mapping array and mRNA expression studies become more cost effective, the number of samples profiled on these platforms will increase. Specialized user-friendly tools for efficient visualization, such as SNPExpress, will therefore be indispensable. In fact, the initial version of SNPExpress has already been successfully applied in showing segmental uniparental disomy as a recurrent mechanism for homozygous *CEBPA *mutations in acute myeloid leukemia [38].

Other tools for visualizing and processing SNP array data, such as SNPScan [39], SIGMA [40], Array Fusion [41], Partek Genomics Suite [42] and GenePattern [43] have been developed. Most of these tools incorporate visualization options for displaying LOH (GenePattern, Partek Genomics Suite, SNPScan) and copy number (all but ArrayFusion), whereas SNPScan and ArrayFusion have output functionality that facilitates linking SNP data to the UCSC genome browser [39,41]. Some are linked to a private database, which restricts pre-processing of the array data, but gives the advantage of data storage [40]. GenePattern and the Partek Genomics Suite provide normalization and data smoothing functionality. These two packages and SNPScan have also incorporated options for combined analysis of paired samples, i.e., tumor and normal. Like SNPExpress, SNPscan, GenePattern, and the Partek Genomics Suite can detect regions of LOH, amplification and deletion. None of these tools describe the ability to process Illumina BeadArray files. Where SNPExpress may lack the opportunity to directly process raw data files (such as Affymetrix CEL-files), it adds integrated visualization of expression (Affymetrix) and DNA copy number and genotype (Affymetrix and Illumina) data. Moreover, we believe that this is provided in a user-friendly way that does not require specialist computer knowledge.

SNPExpress has some limitations. A full-length chromosome view depicting gains, losses and the regions showing LOH is feasible using SNPExpress. However, the large datasets generated by the 500 K mapping array platform makes it impossible to visualize the sequentially aligned SNPs of the full-length chromosomes on one screen. Selecting the most informative SNPs, i.e., representative for particular haplotypes, may solve this issue. Such algorithms are currently in development. Furthermore, the current implementation of the HMM could also be improved by implementing a HMM that takes into account the effects of linkage disequilibrium, i.e., LD-HMM [18]. The number of samples to be visualized concurrently is limited by the memory available to the application.

## Conclusion

The power of SNPExpress, as with previously developed tools [44], is its high accessibility and powerful visualization, which facilitates the identification of biologically and clinically relevant entities. We have shown that recurrent biologically relevant entities, such as chromosomal gains or losses and LOH in AML, are accurately identified with SNPExpress. Hence, SNPExpress will be beneficial to genome-wide studies by providing an integrated view of data from DNA mapping and mRNA expression arrays in an easily accessible and accurate way.

## Availability and Requirements

Project name: SNPExpress

Project homepage: 

(Including downloadable genotype-, copy number-, expression- and HMM copy number example files of two AML patients genotyped with Affymetrix 250 K *Nsp*I DNA mapping array and gene expression profiled with Affymetrix U133Plus2.0 GeneChips)

Operating system: Platform independent

Programming language: JAVA

Other requirements: JAVA 1.5 or higher.

License: The tool is available free of charge. Source code is available upon request.

Any restrictions to use by non-academics: None

## List of Abbreviations

AML: Acute Myeloid Leukemia; PNG: Portable Network Graphics; BRLMM: Bayesian robust linear model with Mahalanobis distance classifier; SNP: Single nucleotide polymorphism; HMM: Hidden Markov Model; CNV: Copy Number Variation; LOH: Loss-of-heterozygosity.

## Authors' contributions

MAS wrote and designed the software; RGWV designed the software, performed the analysis and wrote the manuscript; WGK performed experiments; SA gave intellectual contributions; SH contributed code; PJS gave intellectual contributions; BL gave intellectual contributions; PJMV designed the study, gave intellectual contributions and wrote the manuscript. All authors read and approved the final version of the manuscript.
